# MIDSHIPS: Multicentre Intervention Designed for Self-Harm using Interpersonal Problem-Solving: protocol for a randomised controlled feasibility study

**DOI:** 10.1186/1745-6215-15-163

**Published:** 2014-05-10

**Authors:** Michelle Collinson, David Owens, Paul Blenkiron, Kayleigh Burton, Liz Graham, Simon Hatcher, Allan House, Katie Martin, Louise Pembroke, David Protheroe, Sandy Tubeuf, Amanda Farrin

**Affiliations:** 1 Clinical Trials Research Unit, Leeds Institute of Clinical Trials Research, University of Leeds, Leeds LS2 9JT, UK; 2 Leeds Institute of Health Sciences, University of Leeds, Leeds LS2 9JT, UK; 3 Leeds and York Partnership NHS Foundation Trust, 2150 Century Way, Thorpe Park, Leeds LS15 8ZB, UK; 4 Department of Psychiatry, University of Ottawa, 5457-1145 Carling Avenue, Ottawa, Ontario K1Z 7 K4, Canada

**Keywords:** Self-harm, Problem-solving therapy, Suicide, Self-poisoning, Self-injury

## Abstract

**Background:**

Around 150,000 people each year attend hospitals in England due to self-harm, many of them more than once. Over 5,000 people die by suicide each year in the UK, a quarter of them having attended hospital in the previous year because of self-harm. Self-harm is a major identifiable risk factor for suicide. People receive variable care at hospital; many are not assessed for their psychological needs and little psychological therapy is offered. Despite its frequent occurrence, we have no clear research evidence about how to reduce the repetition of self-harm. Some people who have self-harmed show less active ways of solving problems, and brief problem-solving therapies are considered the most promising psychological treatments.

**Methods/Design:**

This is a pragmatic, individually randomised, controlled, feasibility study comparing interpersonal problem-solving therapy plus treatment-as-usual with treatment-as-usual alone, for adults attending a general hospital following self-harm. A total of 60 participants will be randomised equally between the treatment arms, which will be balanced with respect to the type of most recent self-harm event, number of previous self-harm events, gender and age. Feasibility objectives are as follows: a) To establish and field test procedures for implementing the problem-solving intervention; b) To determine the feasibility and best method of participant recruitment and follow up; c) To assess therapeutic delivery; d) To assess the feasibility of obtaining the definitive trial’s primary and secondary outcomes; e) To assess the perceived burden and acceptability of obtaining the trial’s self-reported outcome data; f) To inform the sample size calculation for the definitive trial.

**Discussion:**

The results of this feasibility study will be used to determine the appropriateness of proceeding to a definitive trial and will allow us to design an achievable trial of interpersonal problem-solving therapy for adults who self-harm.

**Trial registration:**

Current Controlled Trials (ISRCTN54036115)

## Background

Self-harm is usually defined in UK health services as intentional self-poisoning or self-injury, irrespective of motivation [1]. It has been a major health problem in the UK for 50 years. Rates have never been collected for England nationally, but estimates based on the UK Department of Health funded multicentre monitoring project (in the cities of Manchester, Oxford and Derby) suggest that current rates are a little below their highest ever levels seen in 2003: rates in males are around 350 and in females 480 per 100,000 per annum [2]. Hospitals in England deal with an estimated 220,000 episodes undertaken by 150,000 people each year [3]. Around 80% of these hospital episodes are self-poisoning (usually medicines overdose) while 15 to 20% are injuries (most commonly self-cutting). People who self-harm are high users of health and social care services [4] and two-thirds are aged under 35 years [3]. Time-to-event curves based on 11,000 consecutive self-harm episodes in Leeds between October 2004 and September 2007 show evidence for a difference in repetition rates of self-harm between age groups. Those aged between 30 and 59 years are at the highest risk of repetition whilst those aged over 60 are at a reduced risk compared to other age groups (unpublished data, D Owens, R Kelley, A House). Self-harm has a strong link with suicide: 7 per 1000 people die by suicide in the year after attending hospital with a non-fatal episode of self-harm (60 times higher than the general population risk); by 15 years after self-harm, as many as 30 per 1000 people will have died by suicide [1,5-7]. A quarter of the 5000 to 6000 annual suicides in the UK are preceded by a hospital visit due to non-fatal self-harm in the previous year [8]: it is a major identifiable risk factor for suicide. There is also an excess of premature deaths in those with a history of self-harm from natural causes and accidents [9-11], with many life years lost - particularly among young people. Non-fatal repetition is common (1-year repetition rates are 16% or more) [5] and is associated with distress and unresolved interpersonal problems [12].

UK hospital services for people who self-harm are inconsistent; only around half of patients receive a basic assessment of their state of mind and social circumstances before discharge from emergency departments or medical wards [13]. Unfortunately there is no consensus that intervention at this stage is worthwhile and so therapy is not routinely offered. Despite more than 40 randomised controlled trials in self-harm, a Cochrane review [14] found only small and methodologically poor trials had been previously conducted and no clear evidence that intervention reduces repetition. The review suggested potential benefit from small trials involving problem-solving therapy, a brief and focused psychological intervention with a proven benefit in depression that is known to reduce hopelessness and suicidal ideation [15], concluding that ‘larger trials are badly needed’. A comprehensive American review [16] similarly judged the existing studies to have three main limitations: lack of power, poor description of standard care and inconsistent age ranges across studies. It further stated that the ‘trends suggested incremental benefits from some interventions (in particular, problem-solving therapy for people aged 15 or older)’. It is therefore not surprising that the UK’s National Institute for Health and Clinical Excellence (NICE) guideline on self-harm reported that, as far as psychological intervention is concerned, there was ‘insufficient evidence to make any positive or negative recommendations’ [17], a view echoed in a 2010 report from the UK Royal College of Psychiatrists [18].

Trial evidence that post-dates these reviews adds little to guide routine practice. One UK multicentre randomised controlled trial (RCT) of ‘manual-assisted cognitive behavioural therapy’ [19] failed to find any clear benefit from the new treatment, or any indications of how to steer delivery of interventions for self-harm in the UK National Health Service (NHS). It was, however, underpowered for the following reasons: it was restricted to a study sample unrepresentative of the population of people attending hospital because of self-harm; many of the participants in the active treatment arm did not receive the intervention; and the intervention itself was not, on theoretical or empirical grounds, adequately designed for use in self-harm. Two American trials showed benefits with highly selected participants and intensive forms of expert therapy: 1) ten sessions of cognitive therapy reduced non-fatal repetition in people judged to have made suicide attempts [20] and 2) intensive dialectical behaviour therapy reduced repetition of self-harm in young women with a diagnosis of borderline personality disorder [21]. Costs, in terms of time in therapy and therapist expertise, preclude the widespread use of either of these interventions for subgroups of patients in UK hospitals, and the cost-effectiveness of such interventions for self-harm is unknown. A New Zealand trial of problem-solving therapy found that it was effective in preventing repeat presentation to hospital due to self-harm at one year in those who repeat self-harm, but not in those whose index admission was their first episode [22]. In its guidance on the longer term management of self harm, NICE continues to emphasise the need for further research into the modality, duration, and extent of benefit for therapy interventions, carried out in ‘real-world’ clinical settings [23]. A future definitive RCT is planned, however this feasibility study is first required to inform the design of such a large trial. The projected definitive trial will test the hypothesis that brief problem-solving therapy, when compared with treatment as usual, will reduce repetition (whether fatal or non-fatal) of self-harm; the present feasibility study is not designed to test a hypothesis but to pave the way for the successful undertaking of a definitive trial.

## Methods/Design

### Aims and objectives

MIDSHIPS is a pragmatic, multi-centre, individually randomised, controlled feasibility study, designed to determine the practicability of undertaking a definitive RCT of interpersonal problem-solving therapy plus treatment-as-usual (PST + TAU), compared with treatment-as-usual only (TAU), for adults who attend a general hospital due to self-harm.

Objectives relate to the feasibility of implementing a definitive RCT. The content of the manual and procedures for training therapists in PST, recruiting and training therapists, assessing therapist competence, and determining procedures for therapist supervision must be established. The evaluation of processes to screen patients for eligibility, determining feasibility and best methods of recruitment, and establishing participant uptake and retention during follow up must also be examined. It is important to establish the retention rate in therapy, methods for measuring adherence to PST, and characterising the range of TAU offered and attended. It is also essential to assess the feasibility of collecting the primary outcome for the definitive trial (repeat attendance at hospital following self-harm) from hospital records, establishing likelihood of obtaining the main trial’s secondary outcomes (Short Form 36, healthcare resource use, self-reported self-harm) via postal administration or telephone interviews, and assessing the burden and acceptability of self-reported outcome data. Finally, the variability of outcomes must be estimated in order to inform the sample size calculation for a definitive trial.

### Setting

A total of 60 participants (30 in each arm) will be recruited following attendance at a general hospital as a result of self-harm. The three hospitals involved are those whose mental health services are provided by the Leeds and York Partnership NHS Foundation Trust (LYPFT). People not approached to take part in the study can self-refer, provided that they have recently attended the general hospital due to self-harm, through the provision of information about the trial at the Leeds Survivor Led Crisis Service.

### Participants

#### *Inclusion criteria*

To be included in the study, participants must meet the following eligibility criteria: a) be aged 18 years or over; b) have attended the emergency department or been admitted to a general hospital as a consequence of self-harm (intentional self-poisoning or self-injury irrespective of motivation) within the last six weeks; c) not already be participating in the MIDSHIPS trial; and d) where the presenting episode is due to alcohol or recreational drugs, the participant explicitly stated that they were intending self-harm by use of alcohol or recreational drugs.

#### *Exclusion criteria*

Participants will be ineligible for the study if they meet any of the following criteria: a) they are involved in another research project or clinical service involving a conflicting intervention such as the Personality Disorder service; b) they do not live in the LYPFT catchment area; c) they do not provide fully informed written consent; d) they lack the capacity to comply with trial requirements; e) they are not sufficiently proficient in English to contribute to the data collection required for the research; f) there is a perceived risk of violence towards the therapist or researcher; and g) the researcher is unable to contact the participant within eight weeks following the self-harm event.

### Recruitment

Patients attending hospital following self-harm receive a mental health assessment by the clinical staff following physical treatment, during which formal screening for eligibility occurs. Eligible patients are given the MIDSHIPS study card, which briefly describes the study, and are asked to provide written permission for a researcher to contact them. For those who self-refer to the study via the Leeds Survivor Led Crisis Service, the clinical staff review completed study cards, determine eligibility and if appropriate, provide the researcher with the patient’s details.

Following referral, the researcher telephones those who have consented to be contacted to explain the study in more detail and establish full eligibility. The MIDSHIPS participant information sheet is sent to those eligible, who then have at least 24 hours to read and digest the information before the baseline visit, at which written informed consent is obtained.

To inform generalisability of the study results, screening logs are completed for all people screened for entry into MIDSHIPS; for those screened and not recruited, anonymised data are collected on age, gender, reasons for ineligibility and reasons for non-randomisation.

### Randomisation

Consenting participants are randomised on a 1:1 basis to receive PST + TAU or TAU alone via the Clinical Trials Research Unit’s (CTRU) automated 24-hour randomisation system. A computer generated minimisation algorithm incorporating a random element is used to ensure the treatment arms are well balanced for the following characteristics: a) number of previous episodes of self-harm leading to hospital attendance, prior to baseline assessment (one, more than one), b) type of most recent episode (self-poisoning, self-injury, both injury and poisoning combined), c) gender (male, female) and d) age (<30, 30 to 59, ≥60). Participants and therapists are, of necessity, aware of treatment allocation, but the collection of outcome data by researchers is undertaken blind to this knowledge.

### Intervention

#### *Treatment-as-usual*

Treatment following self-harm varies and may involve referral to a multi-disciplinary mental health team, referral to a mental health crisis team and recommendation for attendance at other services including alcohol and drug treatment centres. In many instances in UK current clinical practice, no aftercare is offered, and the only action to follow discharge from hospital is a letter sent to the general practitioner summarising the episode. TAU for randomised participants is recorded in both arms of the study. In order to avoid contamination across the two arms of the trial, we ensured that the therapists did not participate in the TAU activity.

#### *Interpersonal problem-solving therapy*

Overwhelming problems can have an adverse effect on people’s existing problem-solving skills, such that impulsive responding and incomplete solutions may become more likely. In people who have self-harmed, the result may be less active problem-solving, reliance on the actions of others, waiting for resolution, and limited generation of alternative solutions – all of which reinforce existing negative beliefs about self and self-blame. Interpersonal PST is an established, brief, focused, and readily-learned psychotherapy with a proven benefit for depression and is known to reduce hopelessness and suicidal ideation [15]. In a New Zealand RCT of PST, seeking to reduce hopelessness and repetition of self-harm [22], the research team successfully amended the therapy to fit the needs of their patients who had self-harmed. This manual, along with training processes and client workbooks, has been adapted for UK NHS use. Trial therapists have been recruited, as a part-time secondment, from the clinical mental health service in Leeds. The target groups of staff for this recruitment were experienced but not senior practitioners in mental health nursing, occupational therapy, or social work. Training in problem-solving therapy has been delivered as a three-day course followed up with supervision sessions with a senior psychiatrist with experience in self-harm management and problem-solving therapy (A House).

Participants randomised to PST receive up to six one-hour weekly individual sessions delivered one-to-one by the trial therapists. Sessions focus on the identification of current problems and a structured psycho-educative approach to problem-solving. In addition, participants are offered an optional ‘booster’ session, approximately six to eight weeks following the end of therapy.

The therapists receive regular supervision in the use of the problem-solving therapy as part of the research procedure, and participants in the trial remain patients of the clinical service (the single UK National Health Service Trust) from which they are recruited. The therapists can urgently refer any clinical emergencies or concerns about the participant’s mental state to the standard clinical service, to be dealt with promptly through the existing arrangements for urgent assessment and care.

### Data collection

At baseline, the researcher collects details of the index self-harm event (the event leading to hospital attendance which prompted entry into the study), self-harm history, current physical and mental health, social and medical history, current psychotropic medications, education, and employment details. At baseline and at three and six months post-randomisation, the participant is asked to complete the Short-Form 36 [24] and a trial-specific questionnaire about the use of health and social care resources, private expenses, and lost productivity. Self-reported self-harm events are also collected at both follow up time points. All follow up outcome data are collected by post or by telephone following non-response to postal reminders. Participants are sent £10 shopping vouchers with each questionnaire mailing to thank them for their time. On receipt of the six-month questionnaires the researcher telephones the participant to assess the perceived burden and acceptability of the task of completing the research questionnaires, and obtains additional information on treatment received and current psychotropic medications via a trial-specific, semi-structured interview schedule. A further £10 shopping voucher is sent following this interview.

Data relating to therapy are collected directly from therapists and case notes by UK Research Network staff with support from CTRU. These data relate to therapy (type, dates, number of sessions offered and attended and contact between sessions), therapist supervision, referrals to and attendance at other services (including the mental health crisis team and alcohol and drug treatment centres), and psychotropic medication details. Admissions to or attendance at hospital and all-cause mortality are collected from hospital records by the researcher.

### Outcomes

To establish the problem-solving intervention this requires a finalised training manual and successfully implemented procedures for recruiting and training therapists, in addition to having an agreed and field tested method for assessing therapist competence and recording supervision.

Outcomes relating to recruitment methods, uptake and follow up include screening, eligibility, consent, and randomisation rates together with reasons for non-participation and participant retention during follow-up.

To determine whether therapeutic delivery is successful we will examine attendance, therapy completion rates, and reasons for early dropout together with adherence to therapy by participant and therapist. The range of TAU pathways across treatment arms will also be investigated.

Outcomes related to follow up data collection will include the proportion of participants with self-harm repetition data - whether non-fatal or fatal (suicide), a comparison of self-reported self-harm with data collected from hospital records , the proportion of participants with self-reported outcome data and an assessment of missing data.

Statistical outcomes which will inform the sample size calculation for a definitive trial include the repetition of and time to self-harm leading to hospital attendance within six months post-randomisation, and the difference between arms, the variability of self-reported outcomes at follow up and the difference in self-reported outcomes at follow up.

A qualitative summary of burden and acceptability of self-reported outcomes will also be achieved by examining the participant follow-up interviews.

### Sample size

As this is a feasibility study and effectiveness is not being evaluated, formal power calculations are not appropriate. It is recommended that feasibility studies recruit a minimum of 30 participants per arm to allow for the investigation of parameters and outcomes for a definitive RCT [25]. We therefore plan to recruit 60 participants in total, randomised equally between TAU and PST + TAU.

### Statistical analysis

Analysis will take place when the last participant has completed follow up and all available outcome data are received. Analyses will be descriptive in nature and will provide estimates of key parameters for the definitive RCT. No formal statistical testing will take place as the focus will be on confidence interval estimation. All analyses will be conducted on the intention-to-treat population, with participants analysed according to randomised arm, regardless of non-compliance with the protocol or withdrawal from the study.

The feasibility and success of the recruitment strategy will be evaluated by summarising screening, eligibility, consent and randomisation rates, both overall and by centre. Reasons for dropout at each stage will also be examined.

To assess therapeutic delivery, participant attendance will be summarised and will include the number of participants successfully completing the required amount of therapy as determined by the study therapist, the numbers of participants not attending any sessions and the number of and reasons for, early dropouts from therapy. Therapist adherence to the PST manual will be examined by summarising therapy session content via therapist reports and by an independent review of participant notes. TAU data will be examined to enable us to map out the TAU pathway of participants for the definitive trial. Interview data from the PST therapists will be analysed qualitatively and used to refine training and supervision processes.

To inform the sample size calculation for the definitive trial, repetition of and time to self-harm leading to hospital attendance will be summarised by arm at six months post-randomisation. Differences between arms and 95% confidence intervals will be presented. In addition, the proportion of participants with available self-harm data accessed via hospital records will be compared to the proportion of participants with self-reported self-harm.

Self-reported outcome data completion will be assessed by summarising response rates at baseline and follow up. The level of missing data (at the individual item level and for entire outcome measures) together with qualitative participant interview data will inform the appropriateness of the tools and the level of burden for the definitive trial. An exploratory cost-effectiveness analysis (using SF36 and the health and social care resource use questionnaire) will be produced, together with indicative costs for the interventions.

### Trial governance

Ethical approval was granted for this study by the Leeds West Research Ethics Committee (Reference: 12/YH/022). Research governance approval was gained via the National Institute for Health Research Coordinated System for gaining NHS Permissions (CSP 88012). NHS Permission was granted by LYPFT (2011/302/E/L).

The Trial Management Group (TMG), comprising the Chief Investigator, CTRU Team and Co-Investigators, are responsible for the clinical setup, ongoing management, promotion of the study, and interpretation of the results.

An independent Trial Steering Committee (TSC) provides independent and scientific oversight of the study and comprises an independent Chair and five other independent members (including two lay members). A separate Data Monitoring and Ethics Committee is not required for a feasibility study of this nature, rather a subcommittee of the TSC is convened as necessary to review safety issues. The Sponsor (Leeds and York Partnership NHS Foundation Trust) and the CTRU are responsible for ensuring that the study is conducted in accordance with Good Clinical Practice and the Research Governance Framework.

## Discussion

At present, therapy is not routinely offered to people attending hospital due to self-harm as there is no form of therapy that has been proven to be effective [14,16]. The absence of effective treatment is one of the main reasons why fewer than half of the people who come to hospitals in the UK as a consequence of self-harm receive an adequate psychosocial assessment of their needs [13]. Current evidence points towards effectiveness of brief problem-solving therapy but, if it is to become a routinely available treatment in the NHS, we will need clear evidence from a large, multicentre, pragmatic trial in the UK and an economic evaluation; this feasibility study paves the way towards badly needed improvement in patient experience and outcome after self-harm.

The feasibility study has limitations and is by necessity a small undertaking when compared with the definitive trial. For example, Leeds and York mental health services provide for a large urban area (Leeds, England’s fifth largest city), a smaller urban area (York), and a very large rural region in North Yorkshire. The present feasibility study, set up as it is in just one locality (although one reasonably typical of England), will vary from some of the centres that will need to be recruited in a main trial. The main trial will also have more therapists than involved in the present study. We expect that the present study will provide, at reasonable financial and ethical cost, valuable information needed for the planning of the most important aspects of a definitive trial – concerning the therapy manual, recruitment and training of therapists, recruitment and retention of participants, delivery of therapy, adherence to the therapy manual, and the collection of baseline and follow up data.

## Trial status

Recruitment of participants is ongoing in two study centres within LYPFT.

## Abbreviations

CTRU: Clinical Trials Research Unit; LYPFT: Leeds and York Partnership NHS Foundation Trust; NHS: National Health Service; NIHR: National Institute for Health Research; PST: Problem-Solving Therapy; RCT: Randomised Controlled Trial; TAU: Treatment-as-Usual; TMG: Trial Management Group; TSC: Trial Steering Committee.

## Competing interests

The authors declare that they have no competing interests.

## Authors’ contributions

DO is the lead grant holder. DO and AH conceived the study, led on protocol development and contributed to writing this manuscript. AF is a co-holder of the grant, is the statistical guarantor, contributed to the design of the study, protocol development and writing of this manuscript. LG, SH, AH, LP, DP, are co-holders of the grant and contributed to the design of the study, protocol development and writing of this manuscript. ST, KM and PB contributed to the design of the study, protocol development and writing of this manuscript. KB contributed to the design of the study, protocol development, collection of data and writing of this manuscript. MC contributed to the design of the study, wrote the statistical analysis plan and this manuscript. All authors read and approved the final manuscript.

## Authors’ information

DO, AH and SH have been psychiatric clinicians dealing with self-harm and clinical researchers of the topic over many years. SH wrote the therapy manual for use in a trial in New Zealand and AH and LP revised it for the present study. SH is responsible for the training of the therapists and AH for their supervision in the use of the problem-solving therapy. LP has guided the project from a service-user point of view. DP, KM and PB are the key senior clinicians concerned with self-harm in the general hospitals that are recruiting for the study. AF, LG, KB and MC have provided the expertise concerned with the design and conduct of clinical trials. ST is an associate professor in health economics and has expertise in the design and delivery of economic evaluations.
